# Genome-wide association studies of bundle and single fiber length traits reveal the genetic basis of within-sample variation in upland cotton fiber length

**DOI:** 10.3389/fpls.2024.1472675

**Published:** 2024-10-30

**Authors:** Hee Jin Kim, Gregory N. Thyssen, Christopher D. Delhom, David D. Fang, Marina Naoumkina, Christopher B. Florane, Ping Li, Johnie N. Jenkins, Jack C. McCarty, Linghe Zeng, B. Todd Campbell, Don C. Jones

**Affiliations:** ^1^ Southern Regional Research Center, Cotton Fiber Bioscience and Utilization Research Unit, United States Department of Agriculture-Agricultural Research Service (USDA-ARS), New Orleans, LA, United States; ^2^ Sustainable Water Management Research Unit, United States Department of Agriculture-Agricultural Research Service (USDA-ARS), Stoneville, MS, United States; ^3^ Genetics and Sustainable Agriculture Research Unit, United States Department of Agriculture-Agricultural Research Service (USDA-ARS), Mississippi State, MS, United States; ^4^ Crop Genetics Research Unit, United States Department of Agriculture-Agricultural Research Service (USDA-ARS), Stoneville, MS, United States; ^5^ Coastal Plains Soil, Water, and Plant Research Center, United States Department of Agriculture-Agricultural Research Service (USDA-ARS), Florence, SC, United States; ^6^ Agricultural Research, Cotton Incorporated, Cary, NC, United States

**Keywords:** advanced fiber information system, cotton fiber length trait, quantitative trait loci, within-sample variation in cotton fiber length, single fiber length trait

## Abstract

Within-sample variation in cotton fiber length is a major factor influencing the production and quality of yarns. The textile industry has been searching for approaches of improving the long fiber fraction and minimizing the short fiber fraction within a cotton sample to produce superior fiber and yarn quality. USTER^®^ High Volume Instrument (HVI) has been widely used for a rapid assessment of cotton fiber length traits from a fiber bundle. However, its effectiveness for genetic studies has been questioned due to the indirect estimations of the cotton fiber traits that cannot be measured from a fiber bundle. To overcome the limits of the HVI fiber length traits, we utilized the Advanced Fiber Information System (AFIS) measuring fiber length traits directly from individual fibers based on weight or number. Comparative fiber length analyses showed AFIS provided higher sensitivity in detecting the fiber length variations within and among cotton samples than HVI. The weight-based AFIS length traits were strongly correlated with the corresponding HVI lengths, whereas the number-based AFIS mean length showed a relatively weaker correlation with the HVI lengths. Integrations of the weight based-length traits with genome-wide association studies (GWAS) enabled classifying the QTLs specifically associated with long, mean, or short fiber length traits and identified a false positive associated with the indirectly estimated HVI short fiber trait. Unlike the weight based-AFIS length traits, the number-based AFIS length trait did not show a negative correlation with a weight related-HVI property, and identified a single QTL that was not detected by the corresponding HVI trait. These results suggested that integrating the AFIS method with GWAS helped discoveries of the genome loci involved in the within-sample variation in cotton fiber length and characterizations of the fiber length QTLs.

## 1 Introduction

“Within-sample variation in cotton fiber length” which is also often described as “cotton fiber length distribution variability” in the textile industry is a major factor impacting processability at the mill and the quality of spun yarns (Krifa, 2012; Kelly and Hequet, 2018). The variation in cotton fiber length within a sample occurs naturally, and substantially increases during the harvesting and following mechanical processes (Wakeham, 1955; Ayele et al., 2018). The long fiber fraction within a cotton sample affects yarn quality positively, whereas the short fiber fraction has negative effects (Krifa and Ethridge, 2006). Thus, multiple fiber length distribution parameters representing long and short fiber length traits within a cotton sample are used for predicting yarn quality (Cai et al., 2013).

Cotton fiber length parameters can be calculated based on the weight (w) or number (n) of fibers measured (Delhom et al., 2018). Weight-based lengths were traditionally used to avoid the laborious and time-consuming task of counting the number of individual fibers. The availability of automated instruments has expedited the usage of the number-based lengths calculated based on the relative frequency of fibers (Kelly et al., 2015). The textile industry has developed various instruments, including the Suter-Webb sorter, Almeter, High Volume Instrument (HVI), and Advanced Fiber Information System (AFIS) that use different principles of fiber length measurement (Kelly et al., 2015; Delhom et al., 2018). Among them, the HVI has been widely utilized as the primary high-throughput instrument method by textile industries and cotton geneticists for a fast and inexpensive assessment of cotton fiber quality traits (Kelly and Hequet, 2018).

HVI determines three fiber length traits such as upper half mean length (UHML, mm), short fiber index (SFI, %), and length uniformity index (UI, %) (USTER, 2008) from a fiber bundle. UHML represents the fiber length trait of the long fiber fraction within a cotton sample according to the definition, “the mean length-by-number of the longer one half of the fibers by weight” (ASTM, 2020). SFI represents the fiber content of the short fiber fraction within a cotton sample according to the definition “the percentage of fibers shorter than 12.7 mm within a cotton sample” (USTER, 2013). UI is defined as “the ratio between mean length (ML) and UHML expressed as a percentage of UHML” (ASTM, 2020).

Despite the wide acceptance and usage of HVI length traits for cotton genetic studies, HVI was originally designed for classifying the commercial value of cotton bales for the textile industry in 1969 with the basic principles of the fibrograph method to measure bundle fiber properties (Frydrych and Thibodeaux, 2010). The long fiber trait UHML is calculated from the fibrogram that is generated by an optical scan of a fiber beard (USTER, 2008), whereas the short fiber trait SFI is indirectly estimated from the fibrogram by a proprietary algorithm because HVI is unable to measure most short fibers that are hidden within the jaws of the HVI clamp holding at one end of a combed fiber beard (Thibodeaux et al., 2008). The length UI calculated from the two long fiber traits (UHML and ML) without considering the short fiber trait of a cotton sample does not really represent the fiber length UI of an entire cotton sample (Sayeed et al., 2021). Thus, cotton scientists have debated the effectiveness of the HVI method for genetic studies (Kelly et al., 2012).

Advanced Fiber Information System (AFIS) was proposed as a fiber trait measurement method for improving yarn quality through genetic and breeding studies (Kelly et al., 2012) because it was originally designed to predict spinning performance and yarn quality (Shofner et al., 1988). AFIS measures fiber length traits directly from several thousand individual fibers from a cotton sample and determines a complete length distribution based on number or weight (Hinds et al., 2020). The current AFIS version, USTER® AFIS PRO2, uses aeromechanical separation to individualize fibers and electro-optical technology to analyze single fibers captured in an air stream (Kretzschmar and Furter, 2008). AFIS fiber length parameters including upper quartile length-by-weight (UQLw) that is exceeded by 25% of the fiber by weight, two mean length values measured by number (Ln) or weight (Lw), and two short (<12.7 mm) fiber contents measured by number (SFCn) or weight (SFCw) are summarized in **Table 1** (ASTM, 2020).

**Table 1:** Comparisons of HVI and AFIS fiber length parameters.

System		HVI fiber measurement method	AFIS fiber measurement method
Principle		Bundle fibers testing system	Single fiber testing system
Purpose		To evaluate commercial value of bale cotton as a marketing tool	To predict spinning performance and yarn quality
Fiber length traits	Long fiber length	Upper half mean length (UHML) is determined from the fibrogram of a fiber beard.	Upper quartile length-by-weight (UQLw)
	Mean fiber length	Uniformity index (UI, %) is calculated as the ratio of mean length (ML) to the UHML.	Mean length-by-weight (Lw)
			Mean length-by-number (Ln)
	Short fiber (<12.7 mm) percentage	Short fiber index (SFI) is indirectly determined.	Short fiber content-by-weight (SFCw);
			Short fiber content-by-number (SFCn)

Genome-wide association studies (GWAS) have been utilized to detect genetic variations associated with various cotton fiber quality traits of upland cotton (*Gossypium hirsutum* L.), which is the allotetraploid cotton species producing approximately 95% of the cotton fiber used by the textile industry (Yasir et al., 2022). The three HVI length traits (UHML, SFI, and UI) have been extensively utilized for identifying numerous fiber length trait QTLs from the cotton populations composed of natural upland cotton accessions (Fang et al., 2017; Sun et al., 2017; Li et al., 2018; Ma et al., 2018; Li et al., 2020; Song et al., 2021) or recombinant inbred lines (RILs) (Liu et al., 2018; Thyssen et al., 2019; Wang et al., 2022). In contrast, the AFIS single fiber testing method was not frequently used for genetic studies due to the relatively slow processes and extra cost compared with the HVI bundle fiber testing method. More recently, cotton researchers have begun using AFIS fiber quality traits for GWAS analyses (Iqbal et al., 2021; Billings et al., 2022; Li et al., 2024).

Multiple types of plant trait data obtained by various phenotyping methods were often compared and crucially used to identify genetic architectures regulating major traits in other crop plants (Xiao et al., 2022). However, HVI and AFIS fiber quality traits have not been compared for studying genetic architectures controlling the within-sample variation in cotton fiber length due to the complexity of comparing AFIS and HVI fiber length traits that are measured by different principles and definitions. For example, the mean length trait is not included in the final HVI report but is a major length trait in AFIS. The ratio of mean to long lengths is a major length UI trait in HVI but is not determined in AFIS.

In this study, we included the HVI ML values that were originally measured from the HVI fibrogram and classified the HVI and AFIS length traits into three different classes, including 1) long fiber length traits, HVI UHML *vs*. AFIS UQLw; 2) mean fiber length traits, HVI ML *vs*. AFIS Lw & Ln; and 3) short fiber length traits, HVI SFI *vs*. AFIS SFCw & SFCn as summarized in **Table 1**. Comparative GWAS analyses of each class led to identifying and characterizing genome loci associated with the within-sample variation in cotton fiber length determined from a multi-parent advanced generation inter-cross (MAGIC) population composed of a broad range of fiber length properties.

## 2 Materials and methods

### 2.1 Cotton fiber materials and field experiments

A MAGIC population was previously developed from 11 parents that are composed of Acala Ultima, Coker 315, Deltapine Acala 90, Fibermax 966, M240RNR, Paymaster HS 26, Phytogene PSC 355, Stoneville 474, Stoneville 825, Suregrow 747, and Tamcot Pyramid producing various fiber traits (Jenkins et al., 2008; Islam et al., 2016). Detailed information about each parent was described in **Supplementary Table S1**, and the breeding scheme of the upland cotton MAGIC population was summarized in **Supplementary Figure S1**. The eleven parents were crossed in a half-diallel to establish 55 families, which were randomly mated by a bulked pollen approach for five generations and followed by six generations of single seed descent to establish the first 275 RILs (Set A) and the second 275 RILs (Set B) in Starkville, MS, USA as previously described by Islam et al. (2016). In cotton fields of Stoneville, MS in 2014 (STV14, Set A) and 2017 (STV17, Set B), Starkville, MS in 2014 (MSU, Set A), and Florence, SC in 2014 (FLO14, Set A), two replicates of 275 RILs of the MAGIC population along with the 11 parents were grown in two different plots in a randomized complete block design. Each plot was 12 m long with about 120 plants at each year-location. Standard field practices were applied throughout the growing seasons across all years.

### 2.2 HVI bundle fiber property measurement

The ginned fibers were conditioned at 21 ± 1°C and 65 ± 2% relative humidity for 48 h before testing. Bundle fiber properties, including upper half mean fiber length (UHML, mm), short fiber index (SFI, %), and uniformity index (UI, %), were measured by USTER® HVI 1000 located in the Cotton Fiber Testing Lab in USDA ARS-SRRC at New Orleans, LA. ML was calculated from the corresponding UHML and UI with an equation, [UI (%) = ML/UHML]. All instruments for fiber property analyses were properly calibrated according to the manufacturer’s instructions, and standard cotton fibers were obtained from the USDA Agricultural Marketing Service (AMS). A single HVI machine was run with a single comb, and average values of each RIL fiber grown on each field plot were obtained from five individual measurements per sample.

### 2.3 AFIS single fiber property measurements

The preconditioned cotton fibers of each RIL grown on one field plot were used to generate hand-made fiber slivers by an operator manually. Single fiber properties, including upper quartile length (UQLw), mean fiber length-by-weight (Lw), mean fiber length-by-number (Ln), short fiber content-by-weight (SFCw), and short fiber content-by-number (SFCn), were measured by USTER® AFIS PRO and AFIS PRO2 that were commonly calibrated in the Cotton Fiber Testing Lab in USDA ARS-SRRC at New Orleans, LA and Ginning Lab in USDA ARS at Stoneville, MS. The AFIS instruments are included in annual international round trials and are under a service agreement requiring routine examination and calibration checks twice a year by a USTER® technician. The AFIS values of each RIL grown on one field plot were obtained from three slivers with 5000 fibers measured per sliver. Average AFIS data from each RIL fiber sample were calculated from two replicates grown in two different field plots under the same field season.

### 2.4 Phenotype variance analysis and normalization

Fiber length variance and normalization analyses were performed by the method that was previously described by Thyssen et al. (2019). Briefly, raw phenotypic data of each location were separately subjected to ANOVA using PROC MIXED in SAS software version 9.4 (SAS Institute, Cary, NC, USA). Arithmetic means of phenotype values were computed between replicates for GWAS. The best linear unbiased predictor (BLUP) value for each trait of each RIL was calculated across all replicates, years, and locations using the mixed linear model in the R package “lme4” (Bates et al., 2014). The formula was “model = lmer(phenotype ~ (1|line) + (1|location) + (1|year) + 1|(replicate: location):year) + (1|line: location) + (1|line:year))”. The model includes the following random effects: line, location, year, replicate within location and year, interaction between line and location, and interaction between line and year. The fixed effect in the model is the intercept, which is included by default.

### 2.5 DNA isolation and whole genome sequencing

Eleven parental lines and 550 RILs were grown in a greenhouse in New Orleans, LA, USA. Genomic DNA was extracted from the young leaves of ten plants of each line, according to Islam et al. (2016). Young leaves were collected from ten plants of each RIL and bulked. Leaves were stored at −80°C. Total DNA was extracted from the frozen leaves using Omega EZNA^®^ DNA isolation column (Omega Bio-Tek, Norcross, GA). The quality and quantity of DNA were measured using a NanoDrop 2000 spectrophotometer (Thermo Fisher Scientific, Waltham, MA, USA). The extracted DNAs were sent to Novogene Corporation (Chula Vista, CA, USA) for library preparation, and whole genome sequencing using Illumina HiSeq 2500 with paired-end 150 bp reads. For the eleven parental lines, each was sequenced at 20× coverage (about 50 Gb), and for the 550 RILs, each was sequenced at 3× coverage (about 8 Gb).

### 2.6 Genome-wide association study

Sequencing reads of the 11 parents and 550 RILs were aligned to the updated reference genome sequence of upland cotton (Wang et al., 2019) with GSNAP software, as previously described in Wang et al. (2022). The compressed mixed linear model (MLM) marker-trait association analysis was implemented with GAPIT software using the select sequencing variants, input parameter “PCA. total = 3,” and phenotypic data, which was normalized and subsampled as described above (Lipka et al., 2012; Zhang et al., 2010). GAPIT calculated a kinship matrix using the VanRaden method and performed GWAS using the default average clustering algorithm and mean group kinship type (Lipka et al., 2012). In total, 1,548,294 high-quality SNPs (MAF ≥ 0.05) were used to perform GWAS. According to the Bonferroni correction (Liu et al., 2016), the significance threshold of the *p* value for the association was set to 6.45 × 10^−7^ (-log_10_ *p* = 6.19), which was equal to 1/n, where n is the total number of genomic SNPs.

### 2.7 Statistical analyses

Statistical analyses and construction of graphs were performed using correlation, linear regression, and frequency distribution from Prism version 9.5.1 software (Graph-Pad Software, Inc., San Diego, CA). The correlation coefficient value (*r*) was determined by Pearson’s method (Pearson, 1895). Statistical significance was shown at the probability (*p*) levels value under 0.05*, 0.01**, 0.001***, and 0.0001****.

## 3 Results

### 3.1 Comparisons of within-sample variation of cotton fiber length determined by AFIS and HVI

**Figure 1:**
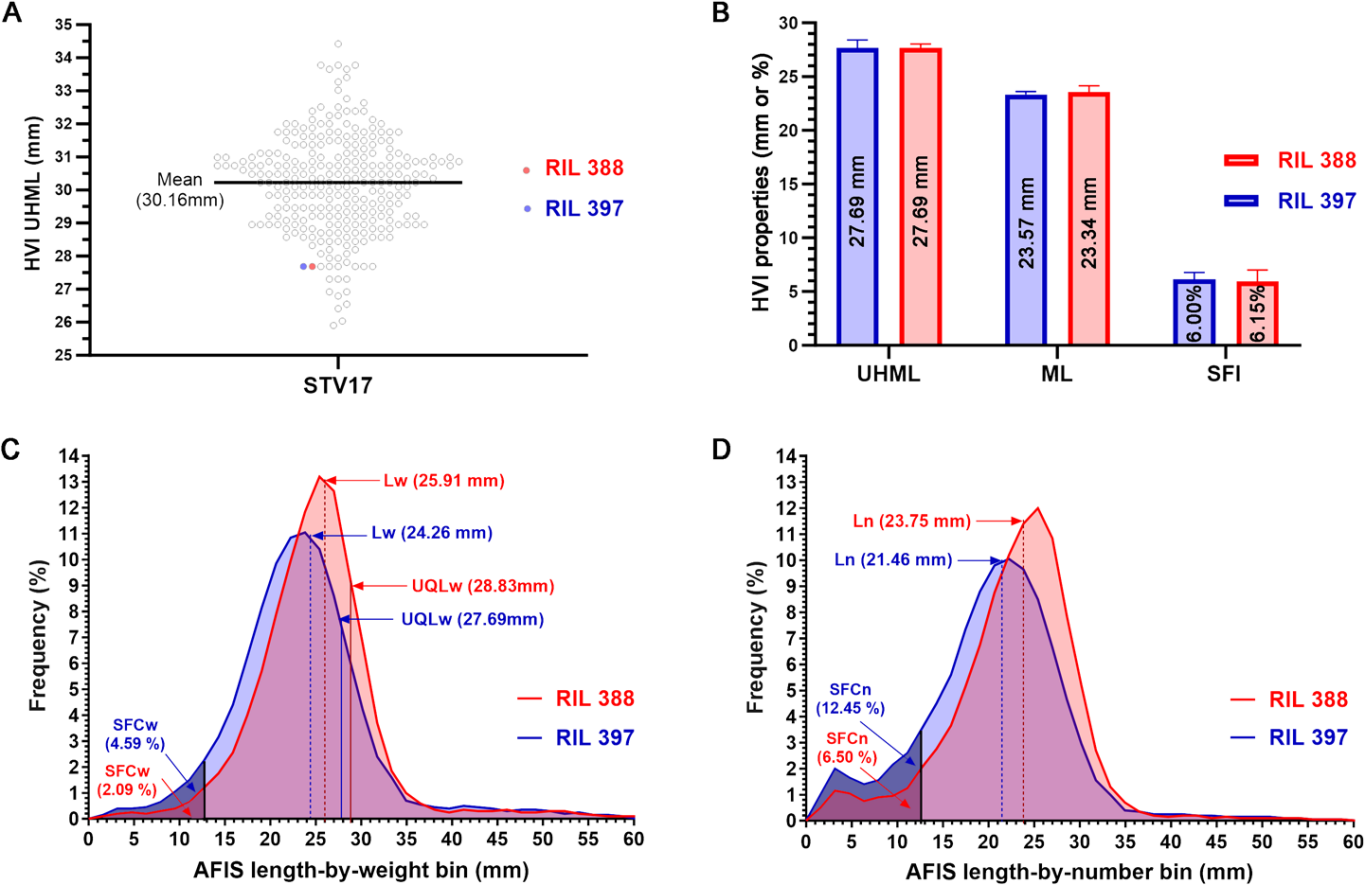
Comparisons of HVI and AFIS fiber length parameters of the two RILs sharing an identical HVI UHML. **(A)** Two selected lines (RILs 388 and 397) sharing an identical HVI UHML (27.69 mm) among the 275 RILs grown at Stoneville, MS in 2017. **(B)** Comparisons of the three HVI fiber lengths representing long, mean, and short fiber traits (UHML, ML, and SFI). **(C)** Comparisons of the weight-based length distributions with the long, mean, and short fiber traits (UQLw, Lw, and SFCw). **(D)** Comparisons of the number-based length distributions with the mean and short fiber traits (Ln and SFCn).

To compare the sensitivity of AFIS and HVI fiber length measurements, we selected two cotton lines (RILs 388 and 397) sharing an identical HVI UHML value with similar standard deviations (27.69 ± 0.36 mm *vs*. 27.69 ± 0.72 mm) (**Figure 1A**). Comparisons of the HVI UHML (*p* = 1.000), ML (*p* = 0.598), and SFI (*p* = 0.880) representing long, mean, and short fiber length traits all showed insignificant differences between the two RILs (**Figure 1B**; **Table 2**).

**Table 2:** Comparisons of representative HVI and AFIS fiber length traits measured from the two RILs sharing an identical HVI UHML.

Method	Length traits	RIL 397^§^			RIL 388^§^			Ratio (%, RIL397/RIL388)	*p* value
		Mean (mm)	SD	N	Mean (mm)	SD	N		
**HVI length**	UHML	27.69	0.72	2	27.69	0.36	2	100.0 ± 2.6	1.000
	ML	23.34	0.27	2	23.57	0.57	2	99.0 ± 1.1	0.598
	SFI	6.15	0.64	2	6.00	1.06	2	103.4 ± 10.7	0.880
**AFIS** **length-by-weight**	UQLw	27.69	0.36	2	28.83	0.54	2	96.1 ± 1.2	0.131
	Lw	24.26	0.18	2	25.91	0.72	2	93.6 ± 0.7	0.088
	SFCw	4.59	0.16	2	2.09	0.16	2	219.6 ± 7.7	0.004**
**AFIS** **length-by-number**	Ln	21.46	0.18	2	23.75	0.54	2	90.4 ± 0.8	0.030*
	SFCn	12.45	0.49	2	6.5	0.1	2	191.5 ± 7.5	0.004**

*p<0.05; **p<0.01; §Two MAGIC RILs sharing the same UHML.

The weight-based AFIS UQLw (*p* = 0.131) and Lw (*p* = 0.088) representing long and mean fiber length traits showed insignificant differences between the two RILs, whereas the weight-based AFIS SFCw representing short fiber length trait showed significant (*p* = 0.004**) two-fold differences between the two RILs (**Table 2**). The distribution curves of the entire AFIS length-by-weight (1.59-60.33 mm) showed visible differences between the two RILs (**Figure 1C**). The comparison of the five components representing the shape and normality of the AFIS length-by-weight distributions confirmed significant (*p* = 0.029*) differences between the RILs (**Table 3**).

**Table 3:** Comparisons of the five components representing the shape and normality of the weight- or number-based AFIS length distribution curves in between the two RILs sharing an identical HVI UHML.

Properties	AFIS length-by-weight		AFIS length-by-number	
	RIL 388	RIL 397	RIL 388	RIL 397
**Peak length (mm)**	25.40	23.81	25.40	22.23
**Peak frequency (%)**	13.20	11.05	12.00	10.05
**Mean length (mm)**	25.91	24.26	23.75	21.46
**Skewness**	1.664	1.436	1.514	1.287
**Kurtosis**	1.475	0.636	0.965	0.303
**^§^Omnibus K^2^**	16.49	12.00	13.59	9.93
**Variation significance**	Yes (p, 0.029*)		Yes (p, 0.015*)	

**^§^** The variance of asymmetry and shape of the two distribution curves was quantified as by D’Agostino-Pearson normality test (D'agostino et al., 1990). The omnibus K^2^ value of a Gaussian distribution is approximately 2. *p<0.05.

The number-based AFIS Ln (*p* = 0.030*) and SFCn (*p* = 0.004**) representing mean and short fiber length traits showed significant differences between the two RILs (**Table 2**). The comparisons of the five components representing the shape and normality of the AFIS length-by-number distributions (1.59-58.74 mm) also confirmed the significant (*p* = 0.015*) differences in the within-sample variation of fiber length between the two RILs (**Figure 1D**; **Table 3**).

In summary, the AFIS single fiber length measurement method provided high sensitivity for detecting the minor length differences between the two RILs whereas the HVI length measurement method did not provide the same sensitivity. The indirectly estimated HVI short fiber trait was significantly different from the directly measured AFIS short fiber traits.

### 3.2 Differential relationships of the AFIS and HVI fiber lengths with HVI MIC property

We further tested the relationships of the HVI and AFIS length traits with the HVI MIC value which is a primary weight-based HVI property measured from a given weight (10 g) of a cotton fiber sample (USTER, 2008; 2013).

**Figure 2:**
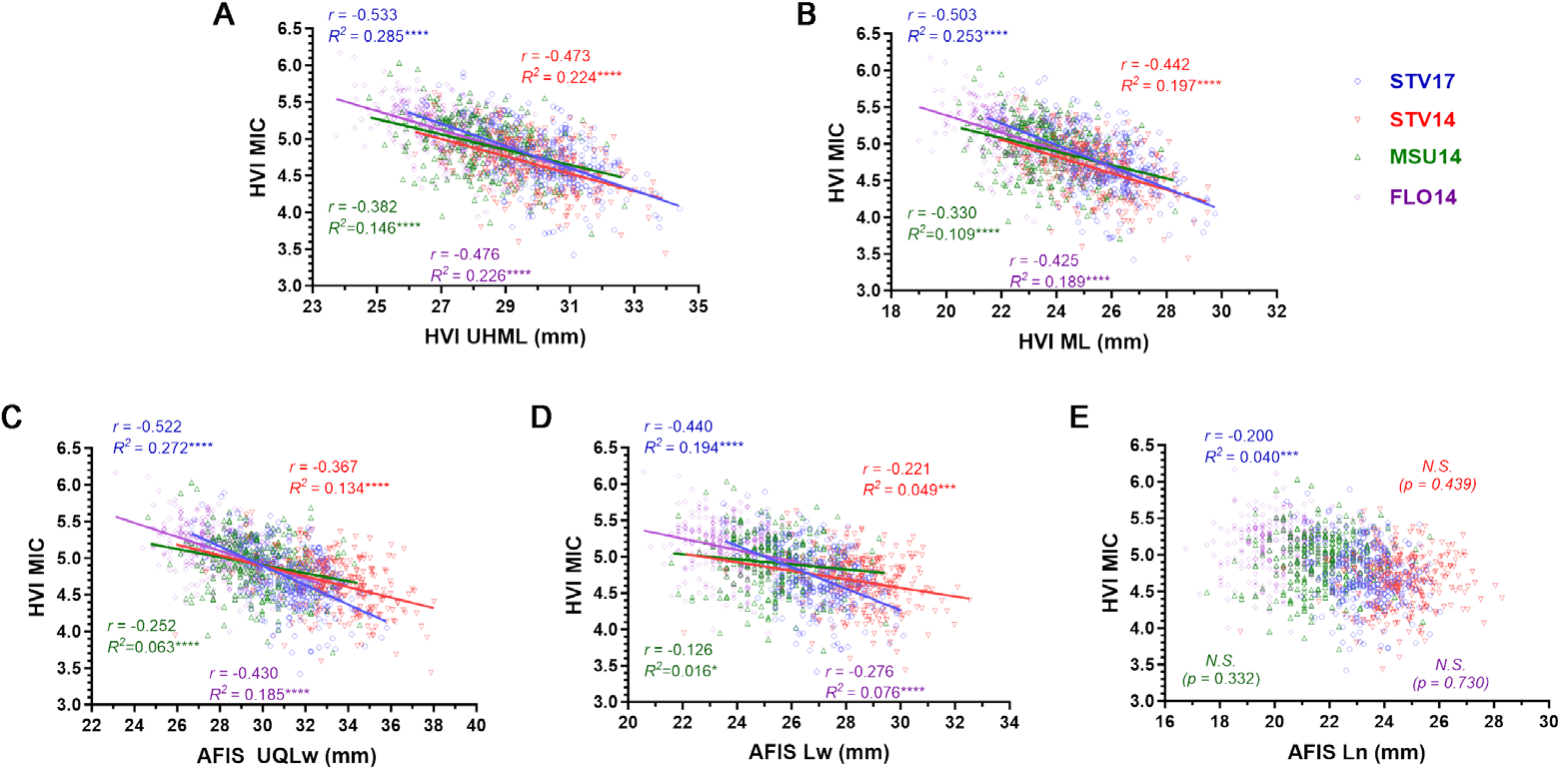
Different relationships of HVI MIC with the five different fiber length parameters. **(A)** HVI UHML *vs*. MIC. **(B)** HVI ML *vs*. MIC. **(C)** Weight-based AFIS UQLw *vs*. MIC. **(D)** Weight-based AFIS Lw *vs*. MIC. **(E)** Number-based AFIS Ln *vs*. MIC. HVI and AFIS properties were measured from 275 MAGIC RILs grown under four different growing conditions, including Stoneville, MS (STV), Starkville, MS (MSU), and Florence, SC in 2014 and 2017. N.S. no significance.

Two HVI fiber lengths (UHML and ML) of the MAGIC populations grown in the four different conditions (STV14, MSU14, FLO14, and STV17) commonly showed significantly negative correlations with the corresponding MIC values (**Figures 2A, B**). The most negative correlation of the MIC values with the UHML (**Figure 2A**, *r* = -0.533) and ML (**Figure 2B**, *r* = -0.503) was commonly detected from the RILs grown in the STV17.

Similarly, the two weight-based AFIS lengths (UQLw and Lw) measured from the MAGIC RILs grown in the four locations also commonly showed significantly negative correlations with the corresponding MIC values (**Figures 2C, D**). The most negative relationship of the MIC with the UQLw (**Figure 2C**, *r* = -0.522) and Lw (**Figure 2D**, *r* = -0.440) was also collected from STV17.

On the contrary, the number-based AFIS length parameter, Ln, collected from STV14 (*p* = 0.439), MSU14 (*p* = 0.322), and FLO14 (*p* = 0.730) showed insignificant relationships with the corresponding MIC values (**Figure 2E**). The Ln collected in STV17 showed a significant but substantially weaker relationship (*r* = -0.200) with the MIC value as compared with four other fiber lengths, including UHML (*r* = -0.533), ML (*r* = -0.503), UQLw (*r* = -0.522), and Lw (*r* = -0.440) collected from the same STV17.

In summary, the weight-based AFIS and HVI fiber length traits (AFIS UQLw, Lw, HVI UHML, and ML) commonly showed negative correlations with the weight-based HVI MIC property, whereas the number-based AFIS fiber length trait (Ln) showed little to no correlation with the weight-based HVI MIC property (**Figure 2**).

### 3.3 Comparisons of HVI and AFIS long fiber length traits for GWAS analyses

**Figure 3:**
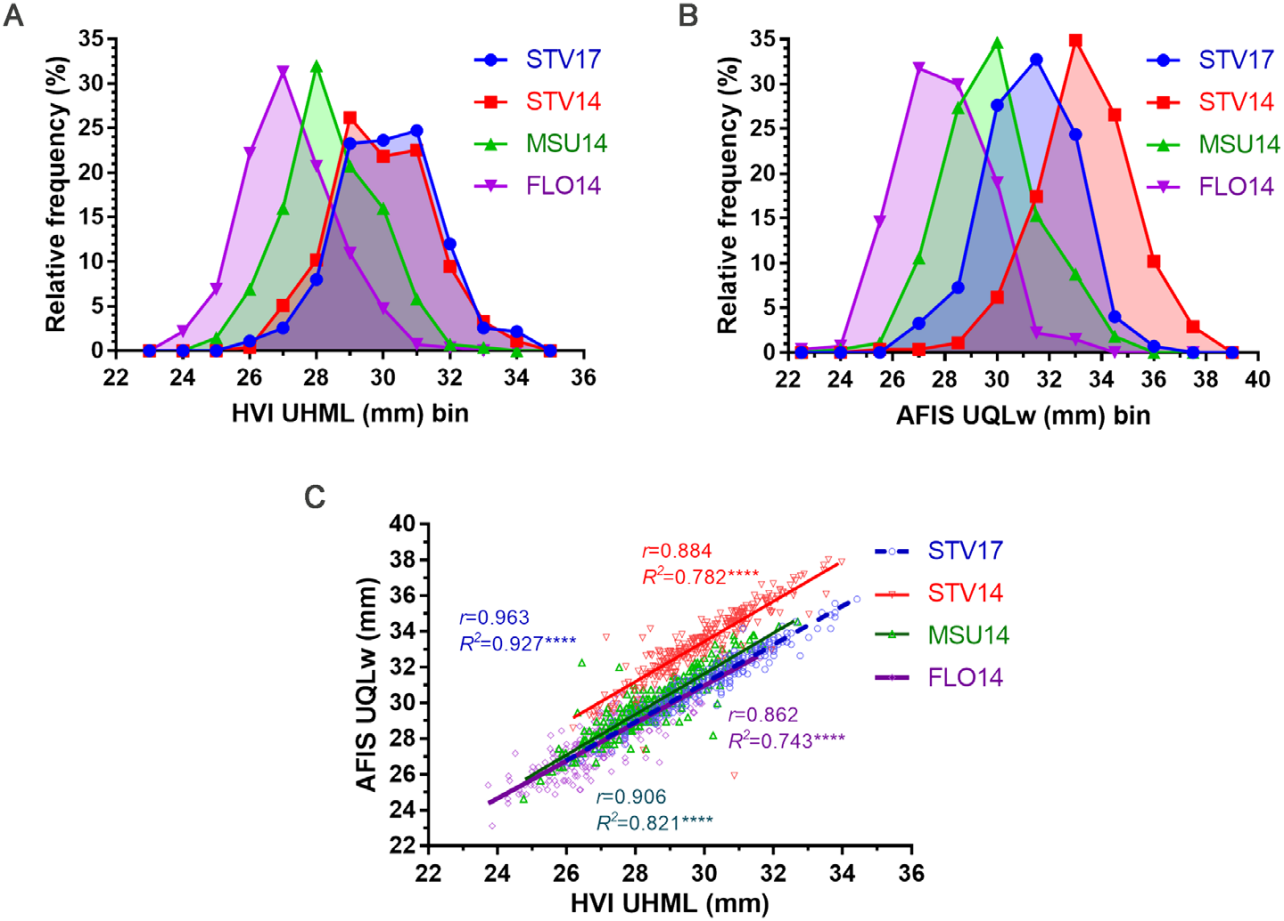
Comparisons of HVI UHML and AFIS UQLw length traits measured from the MAGIC RILs. **(A)** HVI UHML distribution curves. **(B)** AFIS UQLw distribution curves. **(C)** Relationship between HVI UHML and AFIS UQLw. HVI and AFIS properties were measured from 550 MAGIC RILs grown under four different growing conditions, including Stoneville, MS (STV), Starkville, MS (MSU), and Florence, SC (FLO) in 2014 and 2017.

HVI UHML demonstrated wide ranges grown in STV14 (26.19-33.97 mm), STV17 (25.91-34.42 mm), MSU14 (24.76-32.70 mm), and FLO14 (23.73-31.61 mm) as shown in **Figure 3A**. The MAGIC RILs grown at the same location with different seasons (STV14 and 17) showed almost identical HVI UHML distribution curves. The average HVI UHML values of STV14 (29.91 ± 1.46 mm) and STV17 (30.16 ± 1.48 mm) were similar (*p* = 0.188), and significantly (*p* < 0.0001****) greater than those harvested from MSU14 (28.41 ± 1.38 mm) and FLO14 (27.16 ± 1.35 mm) (**Figure 3A**).

The weight-based AFIS UQLw values also showed broad ranges of the MAGIC RILs grown in STV14 (25.92-38.01 mm), STV17 (26.67-35.81 mm), MSU (24.64-34.54 mm), and FLO14 (23.11-32.77 mm) as shown in **Figure 3B**. Unlike the HVI UHML showing similar distributions and average values collected from the different locations, the AFIS UQLw length distributions showed distinct patterns among the four locations. Thus, average AFIS UQLw values showed significant (*p* < 0.0001****) variations with the decreasing order of STV14 (34.34 ± 1.86 mm) > STV17 (31.28 ± 1.66 mm) > MSU14 (29.84 ± 1.72 mm) > FLO14 (27.99 ± 1.65 mm).

The AFIS UQLw collected from STV14 (*r* = 0.884), STV17 (*r* = 0.963), MSU14 (*r* = 0.906), and FLO14 (*r* = 0.862) showed strong and significant (*p* < 0.0001****) linear regression patterns with the corresponding HVI UHML (**Figure 3C**).

**Figure 4:**
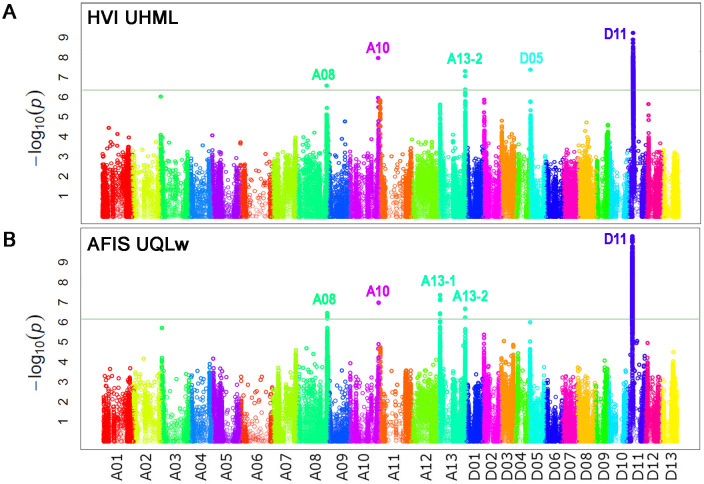
Identifications of the genome loci associated with the two long fiber length traits. **(A)** The Manhattan plot was performed with HVI UHML. **(B)** The Manhattan plot was performed with weight-based AFIS UQLw. GWAS with HVI UHML and AFIS UQLw were performed with the 550 MAGIC RILs grown under four different growth conditions. The vertical axis is labeled with − log(p) values and the significance threshold of p value for the association was set to 6.45 × 10^−7^ (-log_10_ p = 6.19) according to the Bonferroni correction method.

GWAS analyses with HVI UHML values identified five significant peaks located on chromosome (Chr.) A08 at 116 Mb, Chr. A10 at 111 Mb, Chr. A13 at 103 Mb, Chr. D05 at 5.2 Mb, and D11 at 24 Mb (**Figure 4A**; **Table 4**), and named as *qFL-A08*, *qFL-A10*, *qFL-A13-2*, *qFL-D05*, and *qFL-D11* according to the QTL nomenclature proposed by Mccouch (1997). The QTLs identified by HVI UHML in this study overlapped with the HVI fiber quality trait QTLs that were previously identified from the MAGIC RILs by Thyssen et al. (2019) and Wang et al. (2022).

**Table 4:** A list of significant QTLs identified by GWAS with fiber length traits using HVI and AFIS methods.

Fiber length trait	QTL ID	AFIS single fiber property measurement				HVI bundle fiber property measurement			
		Property	Chr.	Peak Position	*p* value	Property	Chr.	Peak Position	*p* value
**Long fiber length trait**	*qFL-A08*	UQLw	A08	115,882,127	3.60E-07	UHML	A08	115,882,127	3.14E-07
	*qFL-A10*	UQLw	A10	111,494,271	1.08E-07	UHML	A10	111,494,271	1.36E-08
	*qFL-A13-1*	UQLw	A13	5,129,350	4.51E-08	-	-	-	-
	*qFL-A13-2*	UQLw	A13	102,608,956	2.14E-07	UHML	A13	102,613,486	8.47E-07
	*qFL-D05*	-	-	-	-	UHML	D05	5,230,213	5.04E-08
	*qFL-D11*	UQLw	D11	24,471,241	4.95E-11	UHML	D11	24,470,231	7.77E-10
**Mean fiber length trait**	*qFL-A10*	Lw	A10	111,494,271	2.08E-07	ML	A10	111,494,271	6.36E-09
	*qFL-A13-1*	Lw	A13	5,129,350	1.00E-08	-	-	-	-
	*qFL-A13-2*	-	-	-	-	ML	A13	102,608,956	1.83E-07
	*qFL-D02*	Lw	D02	4,650,903	8.28E-08	ML	-	-	-
	*qFL-D05*	Lw	D05	5,230,213	2.20E-07	ML	D05	5,230,213	8.16E-08
	*qFL-D11*	Lw	D11	24,389,091	3.08E-08	ML	D11	24,470,231	1.55E-08
	*qFL-A13-1*	Ln	A13	5,125,469	5.39E-09	-	-	-	-
**Short fiber length trait**	*qSF-A05*	SFCw	A05	46,476,311	3.94E-07	-	-	-	-
	*qSF-A07*	-	-	-	-	SFI	A07	91,110,745	2.99E-08
	*qSF-A08*	SFCn	A08	117,571,096	6.55E-07^§^	-	-	-	-

AFIS UQLw identified five significant QTLs (*qFL-A08*, *qFL-A10*, *qFL-A13-1*, *qFL-A13-2*, and *qFL-D11*) from the upland cotton population, as shown in **Figure 4B**; **Table 4**. As predicted from the strong correlations between AFIS UQLw and HVI UHML (**Figure 3C**), four (*qFL-A08*, *qFL-A10*, *qFL-A13-2*, and *qFL-D11*) of the five AFIS UQLw QTLs were also commonly identified by HVI UHML (**Figures 4A, B**). The *qFL-D11* was the most significant peak in both Manhattan plots performed with HVI UHML (**Figure 4A**) and AFIS UQLw (**Figure 4B**). The significance of the *qFL-D11* was greater with AFIS UQLw (*p* = 4.95E-11) than with HVI UHML (*p* = 7.77E-10). Among the five QTLs associated with AFIS UQLw, four (*qFL-A08*, *qFL-A13-1*, *qFL-A13-2*, and *qFL-D11*) are co-located on the HVI UHML QTLs that were previously identified from the MAGIC RILs (Islam et al., 2016; Thyssen et al., 2019; Wang et al., 2022), and one (*qFL-A10*) is overlapped with the HVI strength QTL that was previously identified. The candidate genes and SNPs in the genome loci were summarized in **Supplementary Tables S2**, **S3**.

### 3.4 Comparisons of HVI and AFIS mean fiber length traits for GWAS analyses

**Figure 5:**
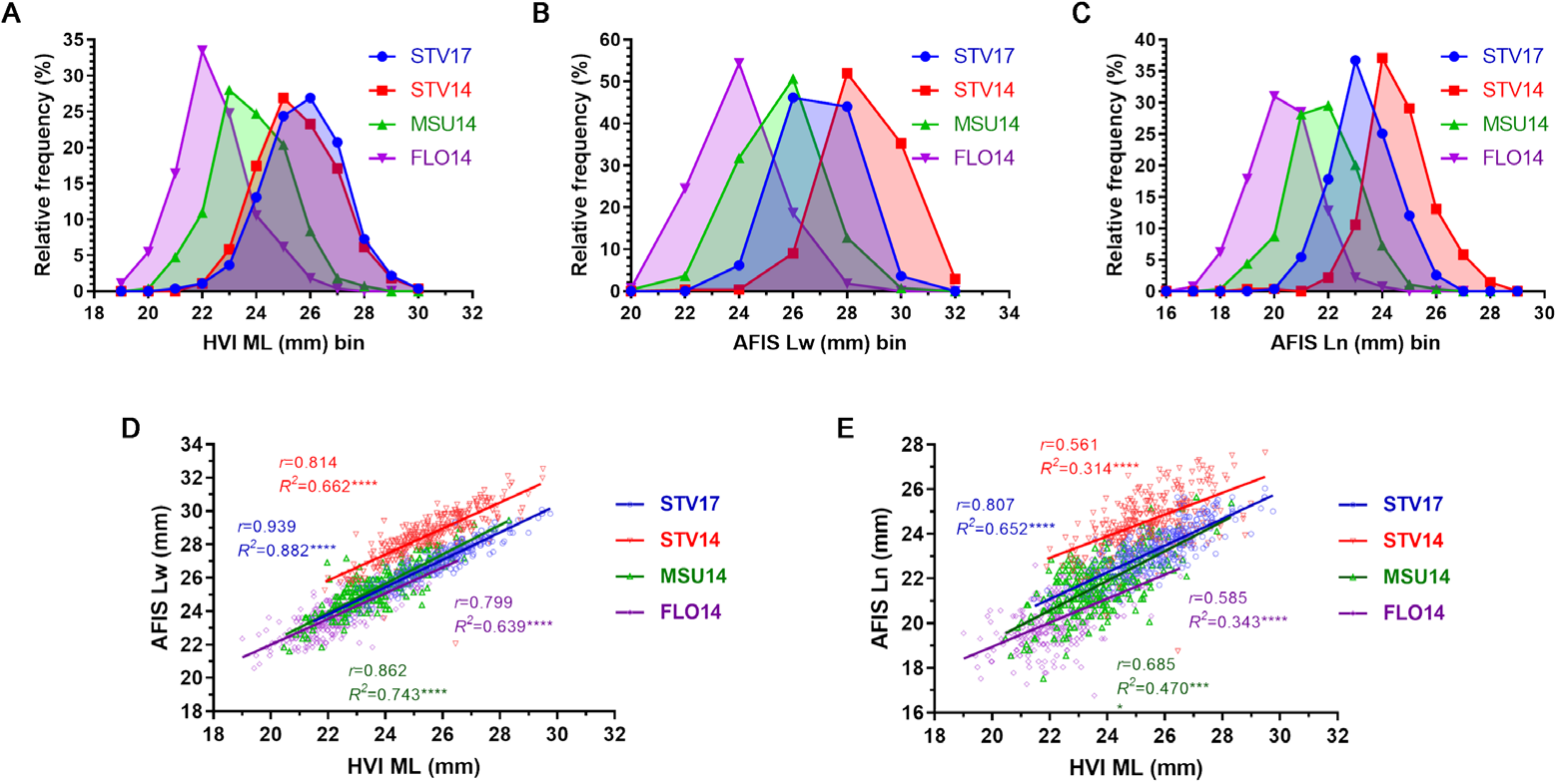
Fiber length comparisons of the three mean fiber lengths measured by HVI and AFIS. **(A)** HVI ML distribution curves. **(B)** Weight-based AFIS Lw distribution curves. **(C)** Number-based AFIS Ln distribution curves. **(D)** Relationship of AFIS Lw and HVI ML. **(E)** Relationship between AFIS Ln and HVI ML. HVI and AFIS mean lengths were measured from 550 MAGIC RILs grown under four different growing conditions, including Stoneville, MS (STV), Starkville, MS (MSU), and Florence, SC (FLO) in 2014 and 2017.

The HVI ML value of each RIL was calculated based on the length UI, which is the ratio of the UHML to the ML (ASTM, 2020). The HVI ML distributions of the MAGIC RILs grown in STV14 (21.88-29.50 mm), STV17 (21.45-29.75 mm), MSU (20.46-28.31 mm), and FLO14 (19.01-26.54 mm) showed wide ranges (**Figure 5A**). The MAGIC RILs grown at the same location with different seasons (STV14 and 17) showed almost identical HVI ML distribution curves. The average ML values of STV14 (25.51 ± 1.43 mm) and STV17 (25.73 ± 1.43 mm) were similar (*p* = 0.238) and significantly (*p* < 0.0001****) greater than those collected from MSU14 (23.78 ± 1.35 mm) and FLO14 (22.45 ± 1.34mm). The average HVI mean fiber length (ML) trait was approximately 4-5 mm shorter than the average HVI long fiber length (UHML) trait.

The weight-based AFIS Lw distributions of the MAGIC RILs grown in STV14 (22.05-32.53 mm), STV17 (23.50-30.10 mm), MSU (19.30-29.46 mm), and FLO14 (20.57-27.69 mm) showed wide ranges of variations (**Figure 5B**). Average AFIS Lw values decreased significantly (*p* < 0.0001****) in the order of STV14 (28.58 ± 1.37 mm) > STV17 (26.86 ± 1.25 mm) > MSU14 (25.44 ± 1.42 mm) > FLO14 (23.89 ± 1.29 mm). The average weight-based AFIS mean fiber length (Lw) trait was approximately 6-8 mm shorter than the average weight-based AFIS long fiber length (UQLw) trait.

Similarly, the number-based AFIS Ln distributions of the MAGIC RILs grown in STV14 (18.75-28.29 mm), STV17 (20.45-26.04 mm), MSU14 (17.53-25.65 mm), and FLO14 (16.76-24.13 mm) also showed broad ranges among the MAGIC RILs (**Figure 5C**). Average Ln values significantly (*p* < 0.0001****) decreased in the order of STV14 (24.63 ± 1.24 mm) > STV17 (23.32 ± 1.06 mm) > MSU14 (21.76 ± 1.30 mm) > FLO14 (20.27 ± 1.23 mm). Both weight- and number-based AFIS measurements enabled distinguishing the distribution curves and average values among the MAGIC RILs grown in the four different locations unlike the HVI measurement (**Figures 5A–C**).

The AFIS Lw values of STV14 (*r* = 0.814), STV17 (*r* = 0.939), MSU (*r* = 0.862), and FLO14 (*r* = 0.799) showed strong and significant (*p* < 0.0001****) linear regression patterns with corresponding HVI ML values (**Figure 5D**). In contrast, the AFIS Ln values of STV14 (*r* = 0.561), STV17 (*r* = 0.807), MSU (*r* = 0.685), and FLO14 (*r* = 0.585) showed relatively weaker correlations with the HVI ML values as compared with the Lw values (**Figure 5E**).

**Figure 6:**
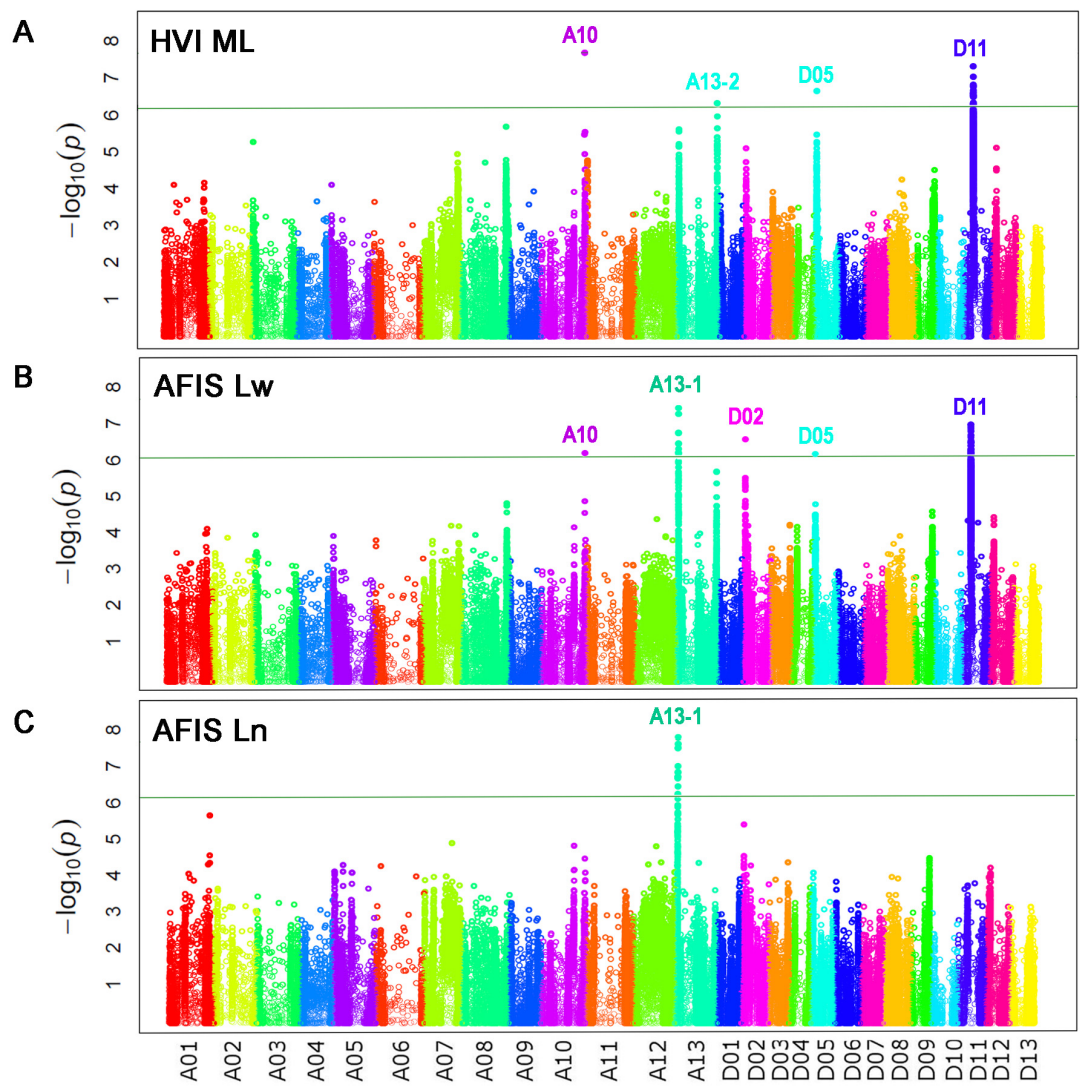
Comparisons of the genome loci associated with three mean lengths measured by HVI and AFIS. **(A)** Manhattan plot for the HVI mean length (ML). **(B)** Manhattan plot for the weight-based AFIS mean length (Lw). **(C)** Manhattan plot for the number-based AFIS mean length (Ln). GWAS were performed with the 550 MAGIC RILs grown under four different growth conditions. The significance threshold of p value for the association was set to 6.45 × 10^−7^ (-log_10_ p = 6.19) according to the Bonferroni correction method.

GWAS analyses with HVI ML identified four QTLs (*qFL-A10*, *qFL-A13-2*, *qFL-D05*, and *qFL-D11*) as shown in **Figure 6A**. Comparisons of the four HVI ML QTLs with the five HVI UHML QTLs showed that the four HVI ML QTLs were commonly associated with both HVI ML and long (HVI UHML) fiber length traits, and the FL-A08 was specifically associated with long (HVI UHML) fiber length traits (**Table 5**).

**Table 5:** Classification of the QTLs associated with within-sample variability of cotton fiber length measured from the MAGIC RILs based on the AFIS fiber length traits.

Class	This study with AFIS & HVI traits					Previous studies with HVI traits^⍑^	
	QTL ID	Chr: Peak	AFIS traits^†^		HVI traits^†^	HVI traits	References^‡^
			w	n			
**Class 1** **Long and mean fiber trait**	*qFL-A10*	Ghir_A10: 111,494,271	UQLw, Lw	–	UHML, ML	STR	Wang et al. (2022)
	*qFL-A13-1*	Ghir_A13: 5,129,350	UQLw, Lw	Ln	–	UHML	Wang et al. (2022)
	*qFL-D11*	Ghir_D11: 24,470,231	UQLw, Lw	–	UHML, ML	UHML	Thyssen et al. (2019), Wang et al. (2022)
**Class 2** **Long fiber trait**	*qFL-A08*	Ghir_A08: 115,882,127	UQLw	–	UHML	UHML	Wang et al. (2022)
	*qFL-A13-2*	Ghir_A13: 102,613,486	UQLw	–	UHML, ML	UHML	Wang et al. (2022)
**Class 3** **Mean fiber trait**	*qFL-D02*	Ghir_D02: 4,650,903	Lw	–	–	–	–
	*qFL-D05*	Ghir_D05: 5,230,213	Lw	–	UHML, ML	UHML	Wang et al. (2022)
**Class 4** **Short fiber trait**	*qSF-A07* ^§^	Ghir_A07: 91,110,745	–	–	SFI	STR, UI, SFI, UHML	Thyssen et al. (2019), Wang et al. (2022)
	*qSF-A05*	Ghir_A05: 46,476,311	SFCw	–	–	–	–
	*qSF-A08*	Ghir_A08: 117,571,096	–	SFCn	–	–	–

**^§^** The qSF-A07 was concluded as a false positive QTL because it was only associated with the indirectly estimated HVI short fiber trait, but not with the directly measured AFIS short fiber traits.

**^‡^** Thyssen et al. (2019) performed GWAS with 473,517 SNPs obtained from the G. hirsutum reference draft genome sequence, whereas Wang et al. (2022) used 1,548,294 SNPs obtained from the updated G. hirsutum reference genome sequence.

^†^n, number-based; Ln, mean length-by-number; Lw, mean length-by-weight; ML, mean length; SFCn, short fiber content-by-number; SFCw, short fiber content-by-weight; SFI, short fiber index; STR, strength; UHML, upper half mean length; UI, uniformity index; UQLw, upper quartile length-by-weight; w, weight-based.

^⍑^qFL-A10 associated with long and mean fiber length traits in this study was previously identified as the QTL related with HVI fiber strength trait by Wang et al. (2022), and qSF-A07 falsely associated with HVI short fiber index was previously identified as a muti-trait QTL associated with HVI strength, UI, SFI, and UHML by Thyssen et al. (2019) and Wang et al. (2022).

The weight-based AFIS Lw identified five significant QTLs (*qFL-A10*, *qFL-A13-1*, *qFL-D02*, *qFL-D05*, and *qFL-D11*) as shown in **Figure 6B**. As predicted from the strong correlations of the AFIS Lw with the AFIS UQLw (**Supplementary Figure S2**), the many QTLs identified by the weight-based AFIS mean length trait (Lw) overlapped with those identified by the weight-based AFIS long length trait (UQLw) and HVI long length trait (UHML) as shown in **Figures 4**, **6B**. However, several QTLs were specifically identified by the AFIS Lw or UQLw. Comparisons of the QTLs identified by the AFIS long (UQLw) and mean (Lw) length traits demonstrating the average fiber length differences of 6-8 mm classified the weight-based AFIS length trait QTLs into three different classes (**Table 5**). Class 1, “*qFL-A10*, *qFLA13-1*, and *qFL-D11*”, were commonly identified by the weight-based AFIS mean and long fiber length traits. Class 2, “*qFL-A08* and *qFL-A13-2*” were specifically identified by the weight-based AFIS long fiber length trait. Class 3, “*qFL-D02* and *qFL-D05*” were specifically identified by the weight-based mean fiber length trait. For the classification of the QTL associated with the within-sample variability of cotton fiber length (**Table 5**), we used the AFIS length traits showing superior sensitivity of fiber length measurements to the HVI length traits. When used both HVI and AFIS long and mean length traits together, the *qFL-A08* was specifically associated with the long fiber length traits measured by both HVI and AFIS, whereas the *qFL-A10* and *qFL-D11* were commonly associated with the long and mean fiber length traits measured by both HVI and AFIS.

The number-based AFIS Ln identified a single peak located on Chr. A13 at 5 Mb (*qFL-A13-1* in **Figure 6C**) that was unique as compared with the multiple QTLs identified by each of the other four length traits, including the HVI UHML (**Figure 4A**), weight-based AFIS UQLw (**Figure 4B**), HVI ML (**Figure 6A**), and weight-based AFIS Lw (**Figure 6B**). The *qFL-A13-1* locus was also significantly identified by the weight-based AFIS UQLw (**Figure 4B**) and AFIS Lw (**Figure 6B**). Still, it was insignificantly associated with the two HVI fiber lengths, UHML (**Figure 4A**) and ML (**Figure 6A**) in this study. The significance of the *qFL-A13-1* locus with AFIS Ln (*p* = 5.39E-09) was substantially greater than that analyzed with AFIS UQLw (*p* = 4.51E-08) and Lw (*p* = 4.51E-08) as shown in **Table 4**. The differences in the QTLs and significance identified between the number-based Ln and weight-based Lw suggested that the principle of fiber length measurement plays a crucial role in identifying the fiber length QTLs. The candidate genes and SNPs of the mean length QTLs were summarized in **Supplementary Tables S2**, **S3**.

### 3.5 Comparisons of HVI and AFIS short fiber length traits for GWAS analyses

To assess the percentage of short fibers (<12.7 mm) within a cotton sample, HVI indirectly estimated the short fiber index (SFI), whereas AFIS determines short fiber contents directly measured from individual fibers by weight (SFCw) or number (SFCn).

**Figure 7:**
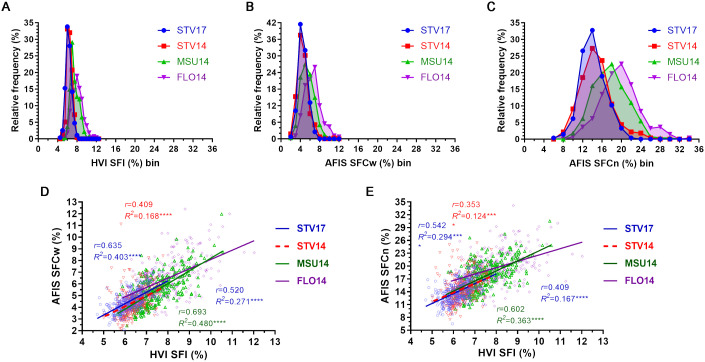
Comparisons of the short fiber traits measured by HVI and AFIS. **(A)** Distribution curves of the indirectly estimated HVI SFI. **(B)** Distribution curves of the directly determined weight-based AFIS SFCw. **(C)** Distribution curves of the directly determined number-based AFIS SFCn. **(D)** Relationship of AFIS SFCw with HVI SFI. **(E)** Relationship of AFIS SFCn with HVI SFI. HVI and AFIS mean lengths were measured from 550 MAGIC RILs grown under four different growing conditions, including Stoneville, MS (STV), Starkville, MS (MSU), and Florence, SC (FLO) in 2014 and 2017.

The HVI SFI distributions showed relatively narrow ranges grown in STV14 (5.01-7.77%), STV17 (4.70-8.00%), MSU14 (5.63-10.57%), and FLO14 (5.82-12.03%) as shown in **Figure 7A**. Average HVI SFI values significantly (*p* = 0.0001****) decreased in the order of FLO14 (8.04 ± 1.08%) > MSU14 (7.49 ± 0.90%) > STV14 (6.44 ± 0.52%) > STV17 (6.24 ± 0.59%).

The weight-based AFIS SFCw demonstrated relatively wider ranges grown in STV14 (2.15-11.11%), STV17 (2.09-7.40%), MSU14 (2.43-11.97%), and FLO14 (3.12-12.40%) than the corresponding HVI SFI ranges (**Figure 7B**). Average AFIS SFCw values similarly decreased in the order of FLO14 (6.52 ± 1.65%) > MSU14 (5.46 ± 1.41%) > STV17 (4.54 ± 0.91%) ≈ STV14 (4.48 ± 1.15%).

The number-based AFIS SFCn of the MAGIC RILs grown in STV14 (7.52-25.44%), STV17 (6.50-21.17%), MSU14 (9.97-30.53%), and FLO14 (10.67-34.17%) was substantially wider than the distribution ranges of both HVI SFI and AFIS SFCw (**Figure 7C**). The average SFCn values decreased in the order of FLO14 (19.67 ± 3.93%) > MSU14 (17.65 ± 3.51%) > STV14 (14.74 ± 3.14%) ≈ STV17 (14.12 ± 2.51%). The average of the number-based SFCn collected from each location was approximately three times more than the corresponding weight-based SFCw.

The directly measured weight-base AFIS SFCw values were moderately correlated with the indirectly estimated HVI SFI values of MSU14 (*r* = 0.693), STV17 (*r* = 0.635), FLO14 (*r* = 0.520) and STV14 (*r* = 0.409) as shown in **Figure 7D**. The directly measured number-based AFIS SFCn values showed relatively weaker correlations with the corresponding HVI SFI values of MSU14 (*r* = 0.602), STV17 (*r* = 0.542), FLO14 (*r* = 0.409), and STV14 (*r* = 0.353) as compared with the weight-based AFIS SFCw (**Figure 7E**). In summary, the indirectly estimated HVI short fiber trait did not show a strong correlation with the directly measured AFIS short fiber trait measured by weight or number.

**Figure 8:**
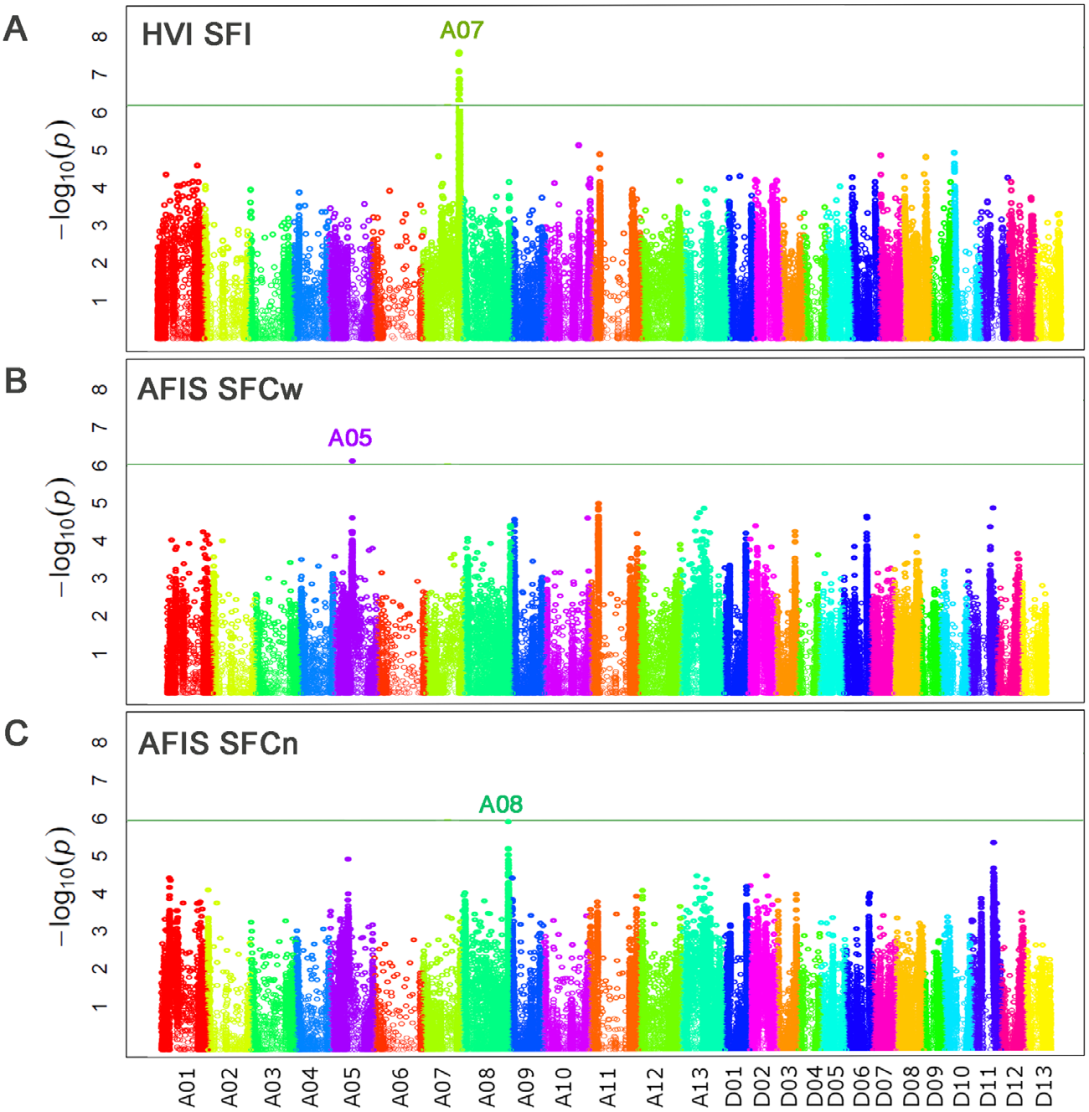
Comparisons of the genome loci associated with three short fiber traits measured by HVI and AFIS. **(A)** Manhattan plot for HVI short fiber index (SFI). **(B)** Manhattan plot for AFIS weight-based short fiber content (SFCw). **(C)** Manhattan plot for AFIS number-based short fiber content (SFCn). The top peak located at chromosome A08 was prominent with a high significance (-log_10_ p = 6.18) but lower than the Bonferroni threshold (-log_10_ p = 6.19).

GWAS analysis with the indirectly estimated HVI SFI identified a single significant (*p* = 2.99E-08) peak (*qSF-A07*) on Chr. A07 at 91 Mb (**Figure 8**; **Table 5**) that was identical to the HVI SFI QTL previously identified from the MAGIC RILs (Islam et al., 2016; Thyssen et al., 2019). In contrast, the *qSF-A07* was not identified by the directly measured AFIS short fiber contents measured by weight and number (**Figure 8**; **Table 5**).

The directly measured weight-based AFIS SFCw identified a significant (*p* = 3.94E-07) genome locus (*qSF-A05*) on Chr. A05 at 46 Mb (**Figure 8B**; **Table 4**), whereas the directly measured number-based AFIS SFCn identified another single prominent peak (*qSF-A08*) on Chr. 08 at 118 Mb (**Figure 8C**; **Table 4**). The significance (*p* = 6.55E-07) of the *qSF-A08* was almost overlapped with but slightly lower than the Bonferroni threshold *p* value (6.45E-07) that has been reported to be too conservative for crop plants (Kaler and Purcell, 2019). The *qSF-A08* region (Ghir_A08: 117.0-117.6 Mb) was overlapped to the *qFL-A08* (Ghir_A08: 110.3-119.6 Mb) region associated with AFIS UQLw (**Supplementary Tables S2**, **S3**). However, the SNP peak of the *qSF-A08* at Ghir_A08: 117,571,096 was different from that of the *qFL-A08* at Ghir_A08: 115,882,127 (**Supplementary Figure S3**).

## 4 Discussion

### 4.1 Comparisons of fiber length traits measured between AFIS and HVI

The MAGIC population composed of the 550 RILs has been suggested to be an ideal system for studying cotton fiber traits due to a broad range of fiber properties as well as no discernible structure or kinship among any of the lines (Islam et al., 2016; Thyssen et al., 2019). Approximately 1.56% of the 1,548,294 high-quality SNPs were heterozygous in the 550 RILs after the five cycles of random mating and six generations of self-pollination via single seed descent (Wang et al., 2022).

Integrations of multiple high-throughput phenotyping methods with GWAS analyses have contributed to the discovery of genetic architecture in other crops in recent years (Yang et al., 2020; Xiao et al., 2022). In the textile industry, HVI and AFIS are two representative high-throughput instruments for assessing cotton fiber quality traits (Kelly and Hequet, 2018). The USTER**^®^** HVI system was developed principally for marketing cotton to the textile industry (Wakelyn et al., 2010), and it is also widely used by the cotton research community for quantitatively analyzing cotton fiber quality traits of large numbers of upland cotton samples.

AFIS has been proposed to be more suitable for cotton genetic and breeding studies than HVI (Kelly et al., 2012). Despite the extra cost and relatively slow process of the AFIS measurement (Delhom et al., 2018), AFIS can provide reliable short fiber length traits as well as long and mean fiber length traits that are all directly measured from individual fibers. In this study, we showed that the AFIS lengths measured from the MAGIC RILs by number and weight both enabled the detection of the length variations more sensitively than the HVI length traits (**Figure 1**). The two-fold differences in the short fiber length traits between the two RILs were detected by both AFIS SFCw and SFCn, which were directly measured but not detected by the indirectly estimated HVI SFI (**Table 2**). Moreover, the variations of the long and mean fiber length traits of the MAGIC grown at the same field with two different seasons (STV14 and 17) were also detected more sensitively by AFIS (UQLw, Lw, and Ln) than HVI (UHML and ML) as shown in **Figures 3**, **5**. As a result, we concluded that the AFIS single fiber testing system provided higher sensitivity in detecting length variations within and among cotton samples as compared to the HVI bundle fiber testing system.

### 4.2 Commonality of the long and mean fiber length QTLs identified by USTER^®^ HVI and weight-based AFIS measurements

Both HVI (UHML & ML) and weight-based AFIS (UQLw & Lw) generated similar patterns of the fiber length traits and GWAS results (**Figures 3**–**6**). These similarities suggest that HVI fiber lengths may also be measured by weight, although the ASTM defines that UHML is measured by a combination of weight and number (ASTM, 2020). Unlike the ASTM, the USTER ***^®^*** company that produces HVI and AFIS instruments defines the USTER ***^®^*** HVI UHML as mean length-by-weight of the longer 50% of fibers (USTER, 2013). The HVI fiber length parameters used in this study were all collected by a USTER^®^ HVI 1000 system and were the weight-based length trait. Thus, the four weight-based fiber length traits measured by USTER^®^ HVI (UHML & ML) and AFIS (UQLw & Lw) commonly showed significantly negative relationships with the primary weight-based HVI MIC property (**Figure 2**). Consistent with the significant relationship of the weight-based length and MIC, the solely significant HVI MIC QTL (Ghir_A13: 102,624,093) overlapped with the weight-based fiber length QTL (*qFL-A13-2*, Ghir_A13: 102,613,486) associated with HVI UHML, HVI ML, and AFIS UQLw (**Supplementary Figure S4**).

Based on the weight-based AFIS fiber length traits showing a greater sensitivity than the USTER^®^ HVI length traits, we classified the seven fiber length QTLs into three different classes composed of Class 1 (*qFL-A10*, *qFL-A13-1*, and *qFL-D11*) commonly associated with both mean and long fiber length traits, Class 2 (*qFL-A08* and *qFL-A13-2*) specifically associated with the long fiber length trait, and Class 3 (*qFL-D02* and *qFL-D05*) specifically associated with mean fiber length trait (**Table 5**). Each region of qFL-A08 (Ghir_A08: 110.3-119.6 Mb), *qFL-A10* (Ghir_A10: 110.0-111.5 Mb), *qFL-A13-1* (Ghir_A13: 5.0-5.5 Mb), *qFL-A13-2* (Ghir_A13: 102.4-102.7 Mb), *qFL-D02* (Ghir_D02: 3.9-4.8 Mb), *qFL-D05* (Ghir_D05: 4.9-5.3 Mb), and *qFL-D11* (Ghir_D11: 24.3-24.8 Mb) encodes 13 to 490 genes as shown in **Supplementary Table S2**. Among those fiber length QTLs, the *qFL-D11* consists of a smaller number of genes with the most significant *p* value than others (**Table 4**, **Supplementary Table S2**), and overlaps with those identified in the previous studies (Ma et al., 2018; Thyssen et al., 2019; Li et al., 2020; Wang et al., 2022). Integrations of SNP data with functional annotation of Arabidopsis orthologs identified thirteen genes within the *qFL-D11* region (**Supplementary Table S4**). Among them, *KIP-related protein 6* (*KRP6*, *Ghir_D11G020340*), *multidrug and toxic compound extrusion transporter* (*MATE*, *Ghir_D11G020400*), and *U2 small nuclear ribonucleoprotein A* (*Ghir_D11G020430*) were abundantly expressed in elongating cotton fibers. KRP6 is a cyclin-dependent kinase inhibitor whose degradation by RING-type E3 ligases affects development and fertility in Arabidopsis (Liu et al., 2008). Ma et al. (2018) suggested *KRP6* (*Gh_D11G1929* corresponding to *Ghir_D11G020340*) as a causal gene in the *qFL-D11* region because its overexpression induced longer and fewer leaf trichomes in Arabidopsis. Consistently, a combination of both transcriptome-wide association study (TWAS) and SNP-based GWAS with a natural cotton population also identified *KRP6* and *U2 small nuclear ribonucleoprotein A* as casual genes associated with fiber length trait in the *qFL-D11* region (Li et al., 2020). On the contrary, a combination of both identity-by-descent (IBD)-based haplotype GWAS and SNP-based GWAS with the MAGIC RILs suggested *MATE* as a causal gene in the *qFL-D11* region (Wang et al., 2015, 2022). Those candidate genes located at the *qFL-D11* still need to be validated by further functional analyses with elongating cotton fibers. Identification of causal genes in other QTLs associated with fiber length trait is complicated due to their wider regions composed of more genes than the *qFL-D11* (**Supplementary Table S2**). Further studies using expression quantitative trait loci (eQTL), biological and computational fine-mapping, and functional analyses may be necessary for narrowing down the region of interest.

### 4.3 Contrasting GWAS results with the mean fiber length traits measured by number and weight

The number-based AFIS Ln identified a single fiber length QTL (*qFL-A13-1*) in contrast to the five QTLs identified by the weight-based AFIS Lw, as shown in **Figure 6** and **Table 5**. The contrasting GWAS results were caused by the differences in the AFIS mean fiber length traits measured by the different principles (number or weight) as shown in **Figures 5D, E**. Unlike the weight-based Lw length showing a strong correlation with the corresponding HVI ML (**Figure 5D**), the number-based Ln length was relatively less correlated with the HVI ML (**Figure 5E**). The number-based AFIS length is directly measured from individual fibers. On the contrary, the weight-based length trait is converted from the number-based fiber length using the linear density that is defined as fiber weight per unit length (tex or g/km) (Kelly et al., 2015). The conversion is performed with the assumption that a uniform linear density exists across all fiber lengths. When the linear density is not consistent across the fiber length, the weight-based length can be biased (Krifa, 2006; Drieling, 2017). Thus, the weight-based AFIS UQLw and Lw calculated by the linear density (**Figures 2C, D**) were significantly correlated with the linear density-dependent MIC values (Montalvo, 2005), whereas the number-based AFIS length (Ln) unaffected by the linear density showed little to no correlations with the linear density-dependent MIC values (**Figure 2E**).

As result, we suggested that the *qFL-A13-1* associated with the number-based length might be a potential candidate to be used for improving fiber length without affecting the weight-related property, whereas the other six QTLs (*qFL-A08*, *qFL-A10*, *qFL-A13-2*, *qFL-D02*, *qFL-D05*, and *qFL-D11*) specifically associated with the weight-based length may be useful to change multiple properties including fiber length, linear density, MIC, and/or their combinations. Further studies will be required to understand how those QTLs are involved in regulating fiber lengths and other traits together.

### 4.4 Comparisons of the three different short fiber trait QTLs identified by indirect or direct measurement method

The high content of short fibers within a cotton sample causes deleterious effects on the quality of spun yarns, so minimizing short fiber content is important for cotton growers and the textile industry (Krifa and Ethridge, 2006). **Table 2** shows that the HVI SFI measurements were not able to detect the two-fold differences in the short fiber contents between the two RILs that were detected by AFIS measurements. The HVI SFI values estimated indirectly from the 550 MAGIC RILs did not show strong correlations with the corresponding AFIS SFCw and SFCn that were directly measured from individual fibers (**Figures 7D, E**). Due to the discrepancy of the short fiber traits measured between the indirect and direct methods, the *qSF-A07* QTL identified by the indirectly estimated HVI SFI was not detected by the directly measured AFIS SFCw and SFCn (**Figure 8**). Although details of the proprietary HVI algorithm are not publicly available, our results support the notion previously suggested by Gipson (1999) and Knowlton (2001) that HVI SFI was indirectly estimated based on HVI UHML, UI, and strength (STR). The *qSF-A07* QTL was also identified by HVI STR and UI with greater significance than SFI from the MAGIC RILs (**Supplementary Figure S5**). The same locus was also previously reported to be associated with multiple HVI fiber quality traits including UHML, UI, STR, ELO, and SFI measured from various upland cotton populations (Islam et al., 2016; Huang et al., 2017; Li et al., 2018; Ma et al., 2018; Dong et al., 2019; Thyssen et al., 2019; Wang et al., 2015; 2022). As a result, we concluded that the *qSF-A07* identified by the indirect HVI SFI estimation was not associated with the real short fiber trait, although the locus may still be involved in other HVI fiber quality traits.

The different principles of measuring AFIS short fiber traits also contributed to identifying different QTLs associated with SFCw and SFCn (**Figures 8B, C**). The weight-based AFIS SFCw measured directly from the MAGIC RILs identified a single QTL (*qSF-A05*), whereas the number-based AFIS SFCn identified another QTL (*qSF-A08*). The difference might be the result of the conversion of the number- to weight-based lengths using linear density, as we discussed in the previous section.

Interestingly, the AFIS SFCn QTL (*qSF-A08*, Ghir_A08: 117.0-117.6 Mb) was located at the cluster consisting of the two other QTLs associated with the fiber length and MIC traits. The fiber length trait QTL (*qFL-A08*, Ghir_A08: 110.3-119.6 Mb) belongs to Class 2, which is specifically associated with the long fiber trait (**Table 5**; **Supplementary Table S2**). The HVI MIC QTL region was previously identified between Ghir_A:08: 108,615,743 and Ghir_A08: 114,514,727 from the MAGIC RILs by Wang et al. (2022). Thus, the *qSF-A08* region overlapped with the QTL regions associated with AFIS UQLw and HVI MIC traits although the SNP peak of the *qSF-A08* at Ghir_A08: 117,571,096 was different from that of the *qFL-A08* at Ghir_A08: 115,882,127 (**Supplementary Figure S3**). The short fiber content of a cotton sample often increases substantially during the mechanical ginning processes that incline to break the long and immature fibers (high UHML and low MIC) into the short fibers (Wakeham, 1955; Anthony et al., 2007). In addition, the threshold value (<12.7 mm) of the short fiber might affect the GWAS results as well. Thus, it is likely that the *qFL-*A08 specifically associated with the long fiber length traits measured by both HVI and AFIS might be the same locus of *qSF-A08* identified by AFIS SFCn.

## 5 Conclusion

Here, we compared two high-throughput fiber quality measurement methods for GWAS analyses of the MAGIC population. The AFIS single fiber measurement method provided higher sensitivity for detecting the within-sample variation in cotton fiber length compared to the conventional HVI bundle fiber measurement method. The principles and methods of measuring fiber length traits crucially affected GWAS results. Integrations of the weight-based AFIS fiber length traits with GWAS enabled the classification of the QTLs specifically associated with long or mean fiber length traits and the identification of a false positive associated with the indirectly estimated short fiber trait. The number-based AFIS length trait may be used to break the negative correlation of fiber length traits with the weight-based fiber properties and potentially improve fiber quality while sustaining cotton production.

## Data Availability

The original contributions presented in the study are included in the article/**Supplementary Material**, further inquiries can be directed to the corresponding author/s.

## Supplementary material

Supplementary Figure 1 Breeding scheme of upland cotton MAGIC population.

Supplementary Figure 2 Relationships of AFIS Lw and AFIS UQLw. The weight-based AFIS mean length (Lw) and long fiber length (UQLw) traits were measured from 550 MAGIC RILs grown under four different growing conditions, including Stoneville, MS (STV), Starkville, MS (MSU), and Florence, SC (FLO) in 2014 and 2017.

Supplementary Figure 3 Comparisons of *qSF-A08* with *qFL-A08*. Manhattan plots of Chr. A08 were determined with AFIS SFCn or HVI UHML of the MAGIC RILs grown in four different seasons and locations. The *qSF-A08* peak was on Chr. A08 at 117,571,096 that was not overlapping with the *qFL-A08* peak on Chr. A08 at 115,882,127.

Supplementary Figure 4 Co-localizations of the A13 locus identified by HVI MIC and the two weight-based fiber lengths. **(A)** Manhattan plot performed with HVI micronaire (MIC). **(B)** Manhattan plot performed with HVI UHML. **(C)** Manhattan plot performed with AFIS UQLw. **(D)** Manhattan plot performed with HVI ML. The significant (*p*, 1.34 x 10^-7^) HVI MIC QTL peaked at Ghir_A13: 102,624,093 was overlapped with the *qFL-A13-2* associated with HVI UHML, AFIS UQLw, and HVI ML shown in **Table 5**. GWAS were performed with the 550 MAGIC RILs grown under four different growth conditions. The significance threshold of p value for the association was set to 6.45 × 10^−7^ (-log_10_ p = 6.19) according to the Bonferroni correction method.

Supplementary Figure 5 The A07 locus identified by HVI multi-traits. **(A)** Manhattan plot performed with HVI short fiber index (SFI). **(B)** Manhattan plot performed with HVI bundle fiber strength (STR). **(C)** Manhattan plot performed with HVI UI. GWAS were performed with the 550 MAGIC RILs grown under four different growth conditions. Vertical axis is labeled with − log(p) values, and the significance threshold of p value for the association was set to 6.45 × 10^−7^ (-log_10_ p = 6.19) according to the Bonferroni correction method.

Supplementary Table 1 Pedigree and origin of the eleven *G. hirsutum* varieties that were used as parents to establish the MAGIC population.

Supplementary Table 2 The candidate genes located at the fiber length QTLs associated with AFIS and HVI cotton fiber length traits (An excel file will be attached).

Supplementary Table 3 SNPs identified with HVI and AFIS fiber length traits.

Supplementary Table 4 Candidate genes located at *qFL-D11*.
