# Supplementary figures and images for: Genome-wide association studies of bundle and single fiber length traits reveal the genetic basis of within-sample variation in upland cotton fiber length
Source: Front Plant Sci. 2024 Oct 30;15:1472675. doi: 10.3389/fpls.2024.1472675 (PMC11571543; doi:10.3389/fpls.2024.1472675)

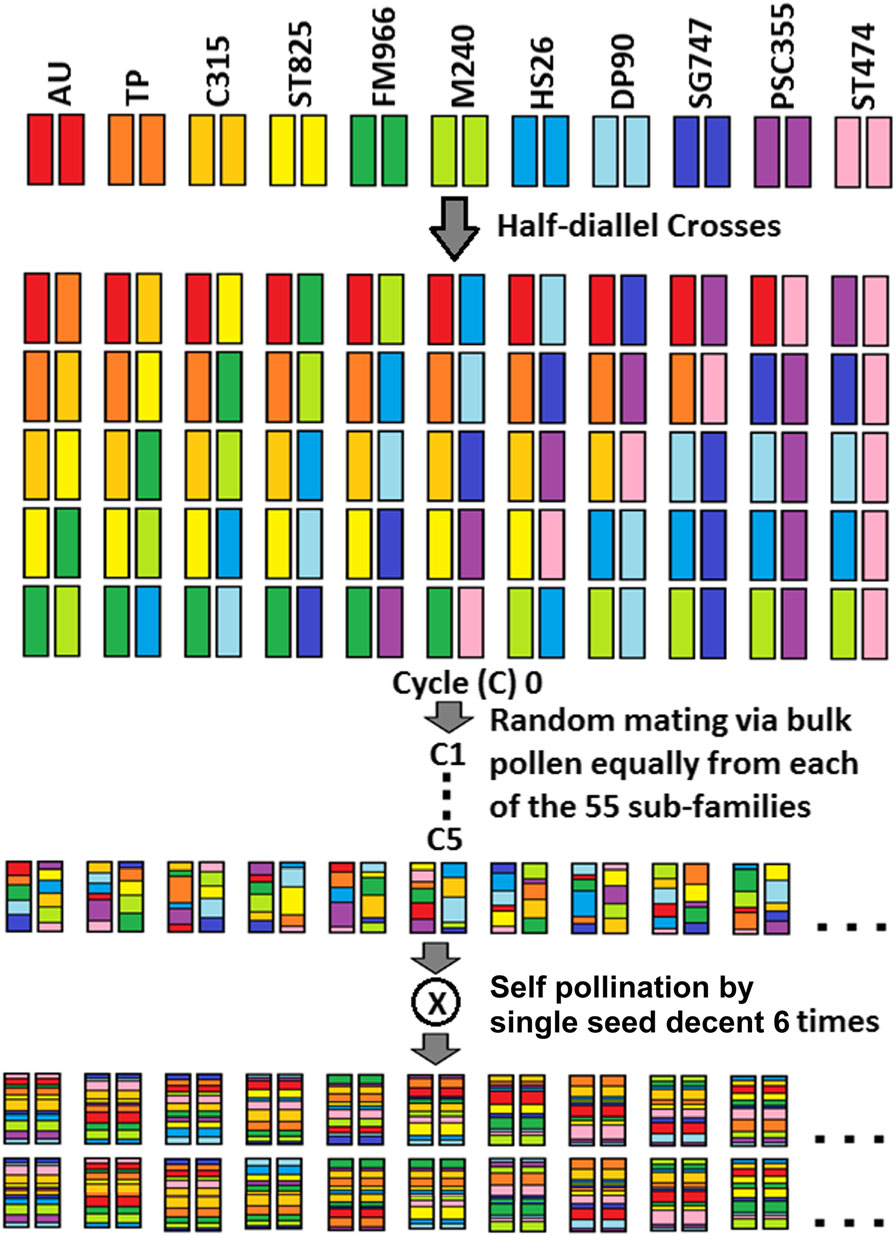

Supplement: Supplementary Figure 1 — Breeding scheme of upland cotton MAGIC population. [file Image1.tif]

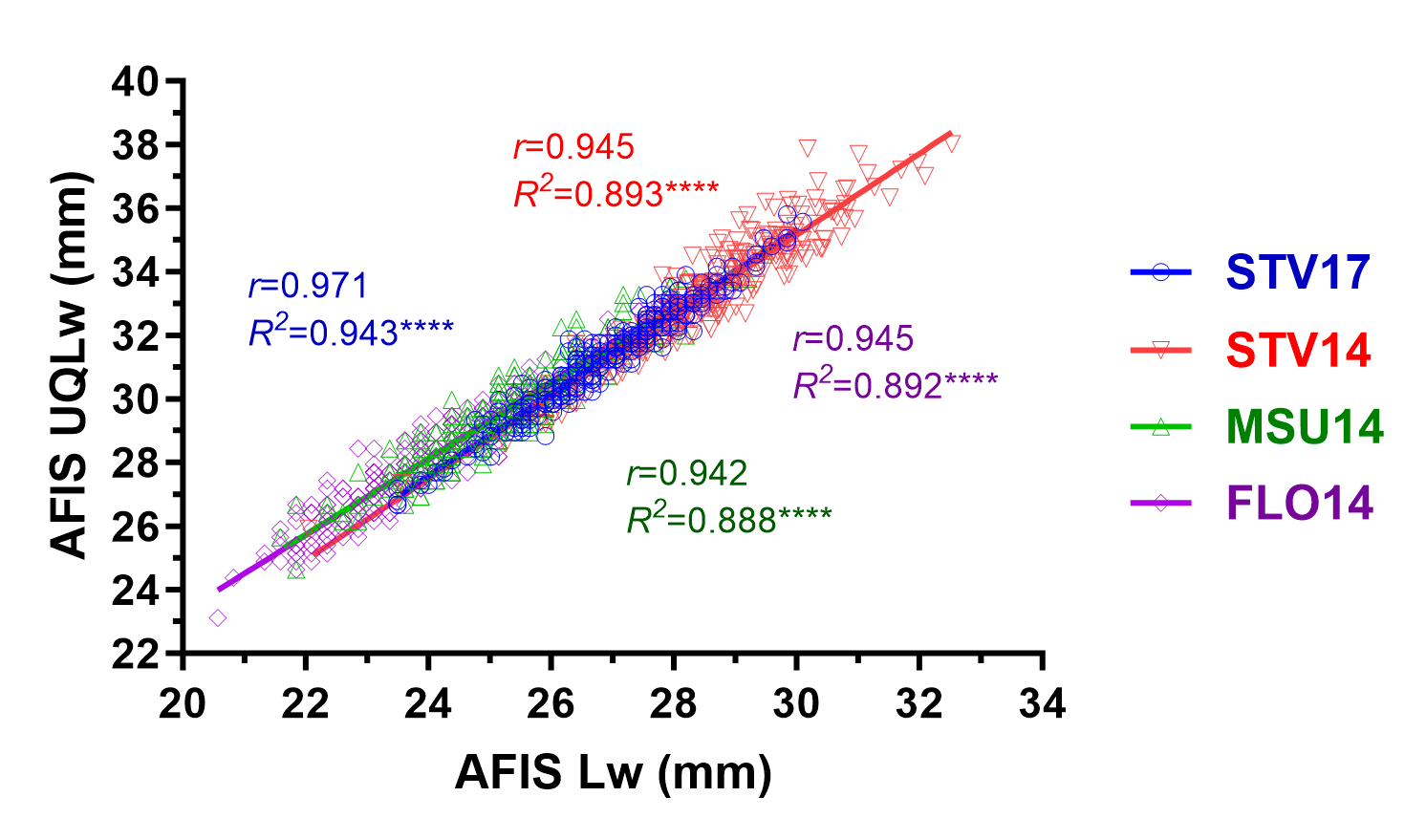

Supplement: Supplementary Figure 2 — Relationships of AFIS Lw and AFIS UQLw. The weight-based AFIS mean length (Lw) and long fiber length (UQLw) traits were measured from 550 MAGIC RILs grown under four different growing conditions, including Stoneville, MS (STV), Starkville, MS (MSU), and Florence, SC (FLO) in 2014 and 2017. [file Image2.tif]

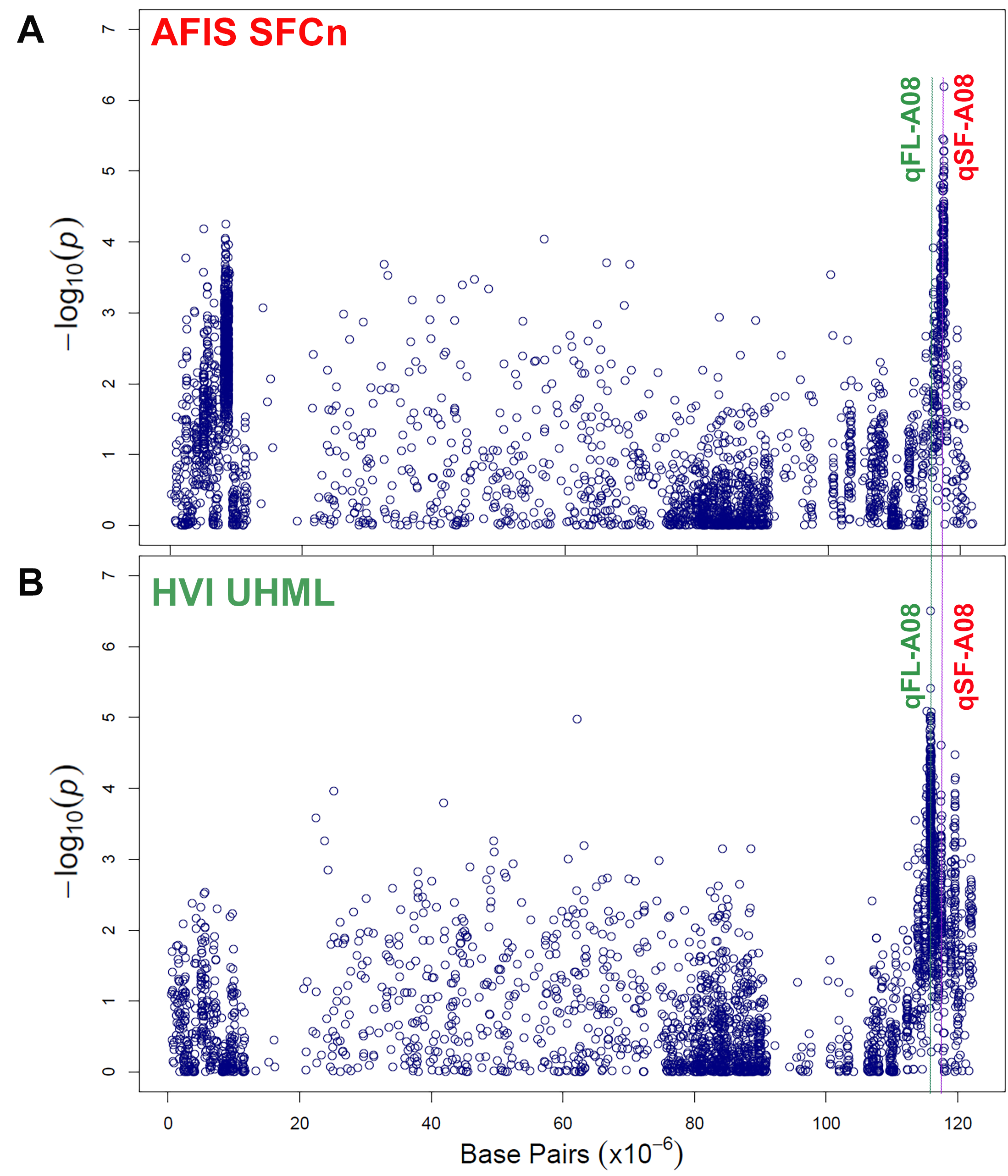

Supplement: Supplementary Figure 3 — Comparisons of qSF-A08 with qFL-A08. Manhattan plots of Chr. A08 were determined with AFIS SFCn or HVI UHML of the MAGIC RILs grown in four different seasons and locations. The qSF-A08 peak was on Chr. A08 at 117,571,096 that was not overlapping with the qFL-A08 peak on Chr. A08 at 115,882,127. [file Image3.tif]

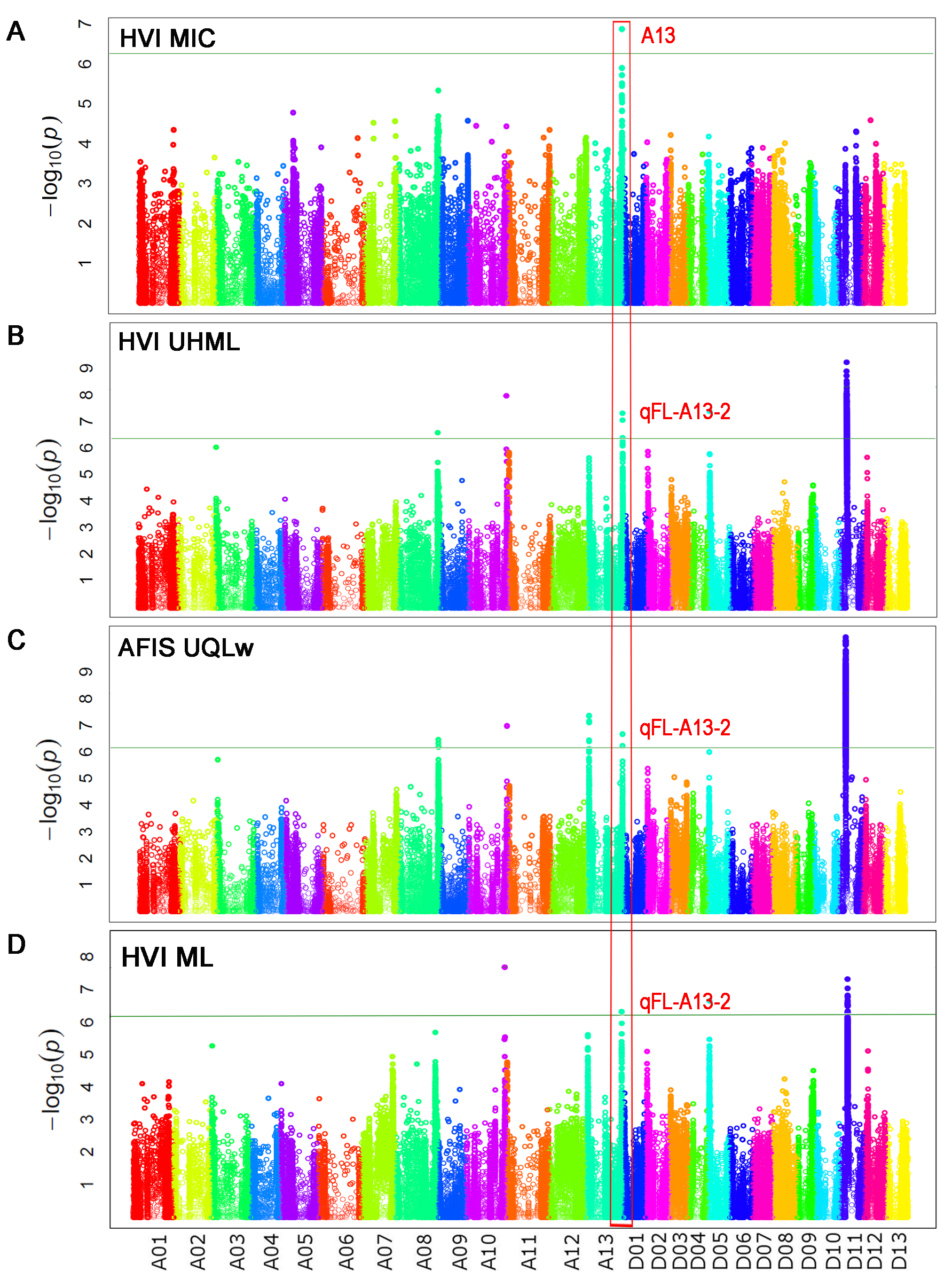

Supplement: Supplementary Figure 4 — Co-localizations of the A13 locus identified by HVI MIC and the two weight-based fiber lengths. (A) Manhattan plot performed with HVI micronaire (MIC). (B) Manhattan plot performed with HVI UHML. (C) Manhattan plot performed with AFIS UQLw. (D) Manhattan plot performed with HVI ML. The significant (p, 1.34 x 10^-7^) HVI MIC QTL peaked at Ghir_A13: 102,624,093 was overlapped with the qFL-A13-2 associated with HVI UHML, AFIS UQLw, and HVI ML shown in Table 5 . GWAS were performed with the 550 MAGIC RILs grown under four different growth conditions. The significance threshold of p value for the association was set to 6.45 × 10^−7^ (-log10 p = 6.19) according to the Bonferroni correction method. [file Image4.tif]

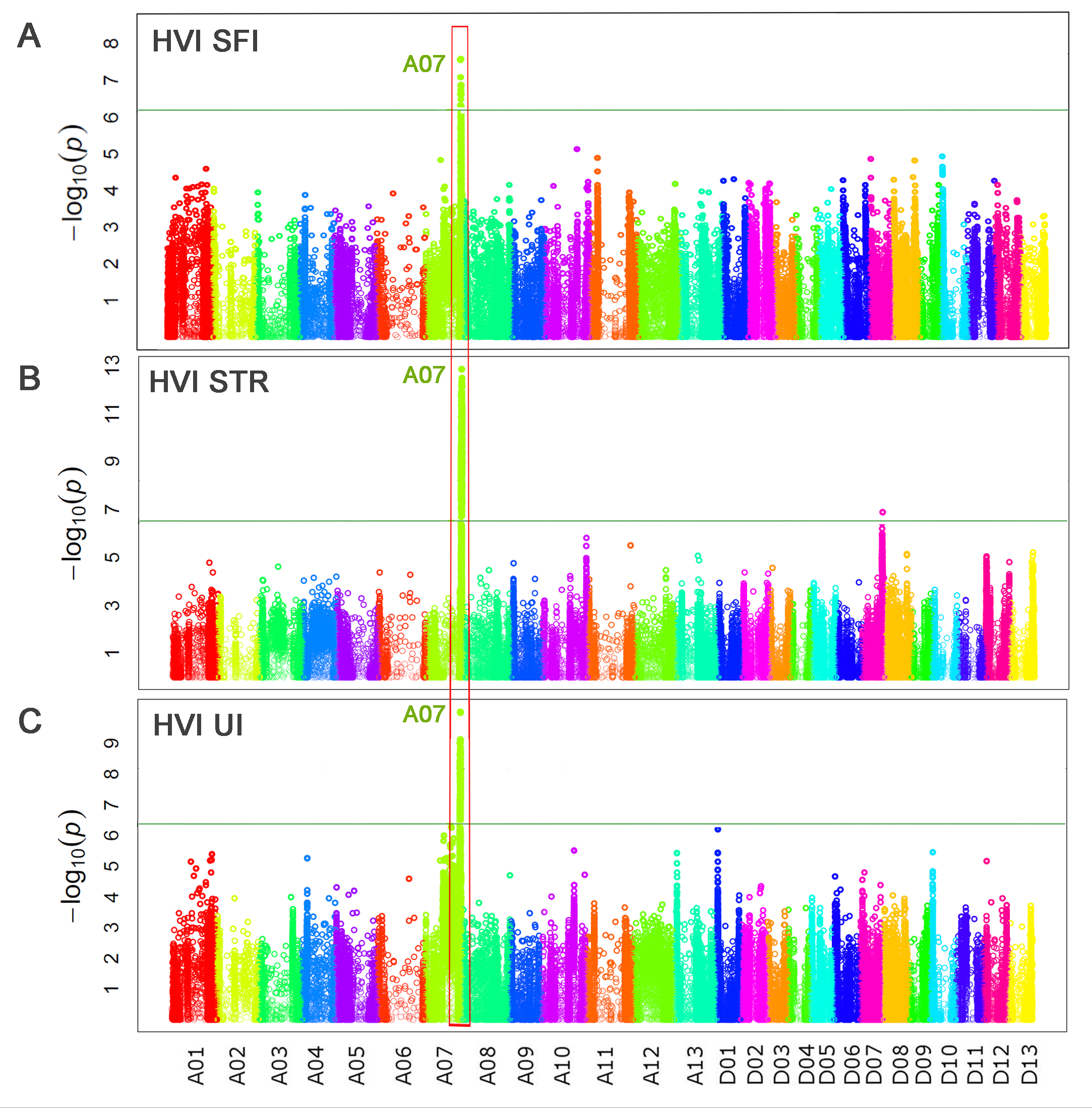

Supplement: Supplementary Figure 5 — The A07 locus identified by HVI multi-traits. (A) Manhattan plot performed with HVI short fiber index (SFI). (B) Manhattan plot performed with HVI bundle fiber strength (STR). (C) Manhattan plot performed with HVI UI. GWAS were performed with the 550 MAGIC RILs grown under four different growth conditions. Vertical axis is labeled with − log(p) values, and the significance threshold of p value for the association was set to 6.45 × 10^−7^ (-log10 p = 6.19) according to the Bonferroni correction method. [file Image5.tif]
